# Supplementary immunization activities (SIAs) in South Africa: comprehensive economic evaluation of an integrated child health delivery platform

**DOI:** 10.3402/gha.v6i0.20056

**Published:** 2013-03-01

**Authors:** Stéphane Verguet, Waasila Jassat, Melanie Y. Bertram, Stephen M. Tollman, Christopher J. L. Murray, Dean T. Jamison, Karen J. Hofman

**Affiliations:** 1 Department of Global Health, University of Washington, Seattle, WA, USA; 2 MRC/Wits Rural Public Health and Health Transitions Research Unit, School of Public Health, Faculty of Health Sciences, University of the Witwatersrand, Johannesburg, South Africa; 3 Health Systems Trust, Johannesburg, South Africa; 4 Umeå Centre for Global Health Research, Umeå University, Umeå, Sweden; 5 INDEPTH Network, Accra, Ghana; 6 Institute for Health Metrics and Evaluation, University of Washington, Seattle, WA, USA

**Keywords:** measles, supplementary immunization activity, child health, integrated delivery platform, cost-effectiveness, sub-Saharan Africa

## Abstract

**Background:**

Supplementary immunization activity (SIA) campaigns provide children with an additional dose of measles vaccine and deliver other interventions, including vitamin A supplements, deworming medications, and oral polio vaccines.

**Objective:**

To assess the cost-effectiveness of the full SIA delivery platform in South Africa (SA).

**Design:**

We used an epidemiologic cost model to estimate the cost-effectiveness of the 2010 SIA campaign. We used province-level campaign data sourced from the District Health Information System, SA, and from planning records of provincial coordinators of the Expanded Programme on Immunization. The data included the number of children immunized with measles and polio vaccines, the number of children given vitamin A supplements and Albendazole tablets, and costs.

**Results:**

The campaign cost $37 million and averted a total of 1,150 deaths (95% uncertainty range: 990–1,360). This ranged from 380 deaths averted in KwaZulu-Natal to 20 deaths averted in the Northern Cape. Vitamin A supplementation alone averted 820 deaths (95% UR: 670–1,040); measles vaccination alone averted 330 deaths (95% UR: 280–370). Incremental cost-effectiveness was $27,100 (95% UR: $18,500–34,400) per death averted nationally, ranging from $11,300 per death averted in the Free State to $91,300 per death averted in the Eastern Cape.

**Conclusions:**

Cost-effectiveness of the SIA child health delivery platform varies substantially across SA provinces, and it is substantially more cost-effective when vitamin A supplementation is included in the interventions administered. Cost-effectiveness assessments should consider health system delivery platforms that integrate multiple interventions, and they should be conducted at the sub-national level.

The World Health Organization (WHO) reported a substantial decrease in global measles mortality, with an estimated 140,000 measles-related deaths in 2010 and a significant 85% decrease from 2000 to 2010 in the African region (1). The WHO strategy to reduce measles mortality includes maintaining high coverage of routine measles immunization and ensuring all children receive a second dose (2). In many low- and middle-income countries, where the second dose is not routinely delivered through primary health care services, this second opportunity is offered during supplementary immunization activities (SIAs) (2) that take place either nationally or sub-nationally (3). This approach was first successfully implemented by the Pan American Health Organization (4). Over the past decade, the strategy has been exported to sub-Saharan Africa and is credited with the recent reduction in measles mortality (1, 5).

As demonstrated in Mexico, a package of interventions can be provided through National Health Weeks (6). SIAs can be expanded to deliver vitamin A supplementation, deworming medicines, oral polio vaccines (OPV) (7), and in malaria endemic countries, insecticide-treated bed nets (ITN) (8, 9). SIAs, which are periodic (usually every 3 years in South Africa) are often integrated into ‘Child Health Days’ (10).

South Africa (SA) has integrated SIAs into polio national immunization days at the provincial level since 1996 (11). Unlike most sub-Saharan African countries, which receive aid, SA's SIAs are entirely funded by the government. The SA campaigns deliver vitamin A supplementation, deworming medicines (Albendazole), and oral polio vaccines, in addition to measles vaccines (MCV). The last poliovirus case in SA was identified in 1989 (12), and measles-related deaths have dropped in SA since the mid-1990s, from about 500 deaths in 1993 to less than 10 in 2007 (13). Prevalence of vitamin A deficiency among 1- to 9-year-olds is prominent, with 14% having a serum vitamin A concentration under 10 µg/L (14). Soil-transmitted-helminth infections among school-aged children remain prevalent in SA coastal regions (15).

Little is known about the cost-effectiveness of the integrated SIA delivery platform (16, 17). Most research to date has focused only on the cost-effectiveness of the measles vaccination component of the platform (18–23). We analyze the cost-effectiveness of the full SIA delivery platform, which we call the child health campaign (CHC) platform. To assess the cost-effectiveness of the *platform*, we look at effectiveness and costs of the interventions included (measles vaccination, polio vaccination, vitamin A supplementation, deworming medicines). Our study takes the entire delivery platform as the unit of analysis. Because budgets are set by provincial authorities and because provinces differ substantially in geography and epidemiology from one another, assessing cost-effectiveness in SA at the provincial level is important.

## Methods

### Effectiveness of the SIA platform

We used coverage data collected for the nine SA provinces in the 2010 CHC, held in the District Health Information System (DHIS), SA. The data included the number of children reached per province by each intervention: measles vaccination for 6-month- to 15-year-olds, polio vaccination for kids aged below 5 years, vitamin A supplementation and deworming for 12–59 months. By intervention and by province, we assessed the burden of disease averted by the one-time 2010 CHC, in deaths averted. Table 1 shows the values of all parameters and references.

**Table 1:** Input values used in the study

Parameter	Value			Sources
	Measles vaccine intervention			
2010 Child Health Campaign coverage of measles vaccine (%)	EC=89 ∣ FS=93 ∣ GP=33^a^ ∣ KZN=95 ∣ LP=98 MP=107^b^ ∣ NC=96 ∣ NW=88 ∣ WC=83			DHIS, SA
Routine coverage of measles vaccine (%) (2003)	EC=76 ∣ FS=70 ∣ GP=71 ∣ KZN=65 ∣ LP=79 MP=67 ∣ NC=72 ∣ NW=69 ∣ WC=77			DHIS, SA
Births cohort (in 1,000s) (2003)	EC=160 ∣ FS=70 ∣ GP=240 ∣ KZN=240 ∣ LP=120 MP=80 ∣ NC=30 ∣ NW=70 ∣ WC=110			DHIS, SA
Case fatality rate from measles	2%^c^			(27,11)
	Vitamin A supplementation intervention			
2010 Child Health Campaign coverage of vitamin A supplementation (%)	EC=89 ∣ FS=90 ∣ GP=100 ∣ KZN=93 ∣ LP=89 MP=91 ∣ NC=53 ∣ NW=68 ∣ WC=75			DHIS, SA
Diarrhea-related deaths among 12- to 59-month-olds	EC=190 ∣ FS=310 ∣ GP=360 ∣ KZN=650 ∣ LP=180 MP=240 ∣ NC=40 ∣ NW=330 ∣ WC=70			(13)
Diarrhea mortality reduction due to vitamin A supplementation	28%			(26)
	Measles-related hospitalizations			
Age-specific probability of hospitalization and length of hospitalization	Age group (years)	Probability of hospitalization	Length of hospitalization (days)	Adapted from (18)
	0–1	0.4	7.2	
	1–4	0.3	6.7	
	5–9	0.1	6.9	
	10–14	0.1	6.0	
	>15	0.1	6.0	
Cost per hospitalization day (2010 US$)^d^		$160		(18)
Cost per outpatient visit (2010 US$)^d^		$23		(18)

a During the campaign, Gauteng only immunized 33% of targeted children with measles vaccine because it had previously implemented a measles immunization emergency campaign in 2009 in response to an outbreak.

b The coverage number surpassing 100% reflects inaccuracies in the denominators of the targeted population.

c 2% corresponds to the lower estimate given by Wolfson and colleagues (27) for South Africa.

d Costs were inflated to 2010 using South Africa Price Index (www.statssa.gov.za/keyindicators/cpi.asp, accessed 6 July 2011).

EC, Eastern Cape; FS, Free State; GP, Gauteng; KZN, KwaZulu-Natal; LP, Limpopo; MP, Mpumalanga; NC, Northern Cape; NW, North West; WC, Western Cape; DHIS, SA, District Health Information System, South Africa.

#### Measles vaccination

We estimated the number of measles deaths averted over 3 years, chosen for the typical 3-year cycle for SA SIAs (3, 5).^1^ We used a Poisson count model relying on national measles-related deaths data from SA's vital registration system for 1993–2007, adjusted for underreporting and completeness, and corrected for misclassified HIV/AIDS deaths; national routine coverage data for the first dose of measles vaccine; SIA's national coverage data (3, 5, 13, 14, 24, 25). The model is as follows:

$$D_{\text{t}}\sim Poisson(\lambda_{\text{t}})$$

2 $$\ln(\lambda_{t})=\beta_{0}+\beta_{1}Cov_{t-7}+\sum_{j=0}^{2}\beta_{j+2}Sia_{t-j}+\ln(Births_{t-7})$$

where *D*_*t*_ counts the measles-related deaths in year *t*, *Sia*_*t−j*_ is the SIA coverage in year *t*−*j* (*j*=0, 1, 2) (measles deaths averted are estimated over three subsequent years because, in recent times in SA, SIAs have been implemented every 3 years), *Cov*_*t*−*7*_ and *Births*_*t*−*7*_ are the routine coverage and birth cohort in year *t*−*7*, respectively. The choice of a 7-year lag provides the best fit to the data (explained in supplementary data, section 1).^2^ The number of measles deaths averted in province *k* is derived as:

$$Deaths_{M,k}=e^{\left(\beta_{0}+\beta_{1}Cov_{k}\right)}(1-e^{\sum_{j\geq 2}\beta_{j}SIA_{k}})Births_{k}$$

where *Cov*_*k*_ is the provincial routine coverage of the first dose of measles, *Births*_*k*_ is the size of the provincial birth cohort, *Sia*_*k*_ is the provincial SIA coverage (DHIS, SA). The number of measles cases averted is calculated by dividing the number of deaths averted by the case fatality rate. Details are provided in the supplementary data (section 1).

#### Vitamin A supplementation

We determined the number of deaths averted by vitamin A supplementation over 1 year (vitamin A campaigns occur annually), using diarrhea-related death estimates from the Global Burden of Disease study for SA (13). Vitamin A supplementation reduces diarrhea-related mortality in 6- to 59-month-olds, shown in the results of seven randomized controlled trials (RCTs) and cluster RCTs reported in a systematic review (RR=0.72; 95% CI: 0.57–0.91) (26). In a given province *k*, we determined the number of deaths averted as a function of number of individuals reached, pre-existing diarrhea deaths among 12–59 month-olds, and reduction of diarrhea mortality due to vitamin A supplementation as documented by RCTs. Details are provided in the supplementary data (section 2).

#### Polio vaccination and deworming

The polio vaccination averted no cases, making the impact of the intervention null (details are provided in the supplementary data, section 3). For anti-helminthics, the SA Medicines Control Council did not give distribution approval in time for the 2010 CHC, so most provinces left out deworming. Consequently, very few children were reached, the burden of worm disease averted was tiny, and impact of the intervention was disregarded in the estimates that follow (details are provided in the supplementary data, section 4).

Each death averted was estimated to correspond to 31 Disability-Adjusted Life Years (DALYs) (discounted at 3%). Years of Life with Disability were disregarded in the calculations, as they represented a tiny fraction of the total DALYs (details are provided in the supplementary data). All the health gains were in Years of Life Lost (none in Years of Life with Disability), hence results are reported in deaths averted.

### Costs

Table 2 shows the costs incurred by the 2010 CHC per province by item categories. Data were obtained from the Expanded Programme on Immunization coordinators. In preparation for the campaign, each province presented a pre-campaign micro-planning document to the National Department of Health, including a budget for campaign items. The items were categorized as follows: vaccines and medicines (MCV and OPV doses were $0.6 and $0.4, respectively), injection materials and consumables, cold chain equipment, waste management supplies, transport, social mobilization materials, training, campaign personnel (registered nurse and other health worker salaries were $109/day and $70/day, respectively). There were gaps in the province budget data; therefore, costs were not available for all items. We used the more complete data available for some provinces (Eastern Cape, Gauteng, KwaZulu-Natal, Limpopo, Mpumalanga, and Western Cape) and extrapolated them for the provinces with missing data (Free State, Northern Cape, and North West). The unit of extrapolation is the number of clinics in the province for some elements (e.g. cold chain equipment) and the number of vaccine doses administered for others (e.g. injection material). Cost data were recorded in 2010 SA Rand (ZAR) then converted to US$ with 1 ZAR=0.14 US$ (Google Finance, accessed 19 July 2011).

**Table 2:** Selected costs (in 2010 1,000 US$) incurred by the 2010 Child Health Campaign by province and nationally

	EC	FS	GP	KZN	LP	MP	NC	NW	WC	South Africa
Measles vaccine doses	1,438	527	1,512	1,952	1,151	743	211	619	796	8,949
Oral polio vaccine doses	40	51	40	481	274	428	118	384	477	2,321
Vitamin A 200,000 IU^a^	0	0	0	0	0	0	0	0	0	0
Albendazole^a^	0	0	0	0	0	0	0	0	0	0
Injection materials	336	119	353	456	269	174	48	139	186	2,080
Cold chain equipment	4	1	2	4	2	2	1	0.1	2	21
Waste management	11	5	18	46	27	16	18	13	18	173
Transport	9	3	5	9	5	4	2	4	4	46
Social mobilization	231	23	36	37	160	24	18	34	83	646
Personnel	6,176	1,056	2,485	4,912	2,885	1,355	64	1,072	1,519	21,524
Others	181	64	185	244	146	89	35	84	104	1,099
Total	8,426	1,849	4,636	8,141	4,919	2,835	515	2,349	3,189	36,859

a Vitamin A and Albendazole were procured for the South African government free of charge.

EC, Eastern Cape; FS, Free State; GP, Gauteng; KZN, KwaZulu-Natal; LP, Limpopo; MP, Mpumalanga; NC, Northern Cape; NW, North West; WC, Western Cape.

IU, international unit.

We estimated the costs avoided due to measles hospitalizations averted, calculated by taking into account age-specific probability of complications, length of hospitalizations, and related direct costs (Table 1). Each patient not hospitalized was assumed to have one outpatient visit. No costs were averted by vitamin A supplementation because vitamin A supplementation was not found to reduce the number of hospitalizations (26).

### Cost-effectiveness

Net health benefits and costs are estimated over 3 years. The 2010 CHC added to pre-existing levels of measles vaccination and vitamin A supplementation interventions. The baseline scenario to which the 2010 CHC platform is compared is the scenario where the 2010 CHC platform is not implemented: no supplemental measles vaccine, vitamin A supplementation, polio vaccines, or deworming medicines were distributed. We defined an incremental cost-effectiveness ratio (ICER) in dollars per death averted for the CHC in each province as *ICER*=*NC / (Death*_*M*_+*Death*_*V*_ *)*, where *NC* is the total net cost, and *Death*_*i*_ is the deaths averted by the intervention of measles vaccination (M) or vitamin A supplementation (V). We defined one ‘hypothetical’ sub incremental cost-effectiveness ratio, *ICER*_*M*_=*NC* */Death*_*M*_, where *NC*_*M*_ includes the net programmatic costs associated with CHC implementation (transport, social mobilization, personnel, others categories in Table 2) and the measles vaccination intervention (MCV, injection materials, cold chain equipment, waste management categories).

At the real exchange rate, SA's GDP per capita is $7,274 (International Monetary Fund, www.imf.org/external/pubs/ft/weo/2011/02, accessed 8 November 2012). Therefore, the CHC platform would be qualified cost-effective, if the ICER is <$7,274 per DALY.

### Sensitivity analysis

We conducted a Monte Carlo multivariate sensitivity analysis to estimate aggregate uncertainty from model inputs. Key parameters were given values, using specific distributions capturing uncertainty simultaneously in 10,000 iterations (details are provided in the supplementary data, section 5). This allows the determination of 95% uncertainty ranges.

Analyses were conducted with the R statistical package (www.r-project.org).

## Results

**Fig. 1:**
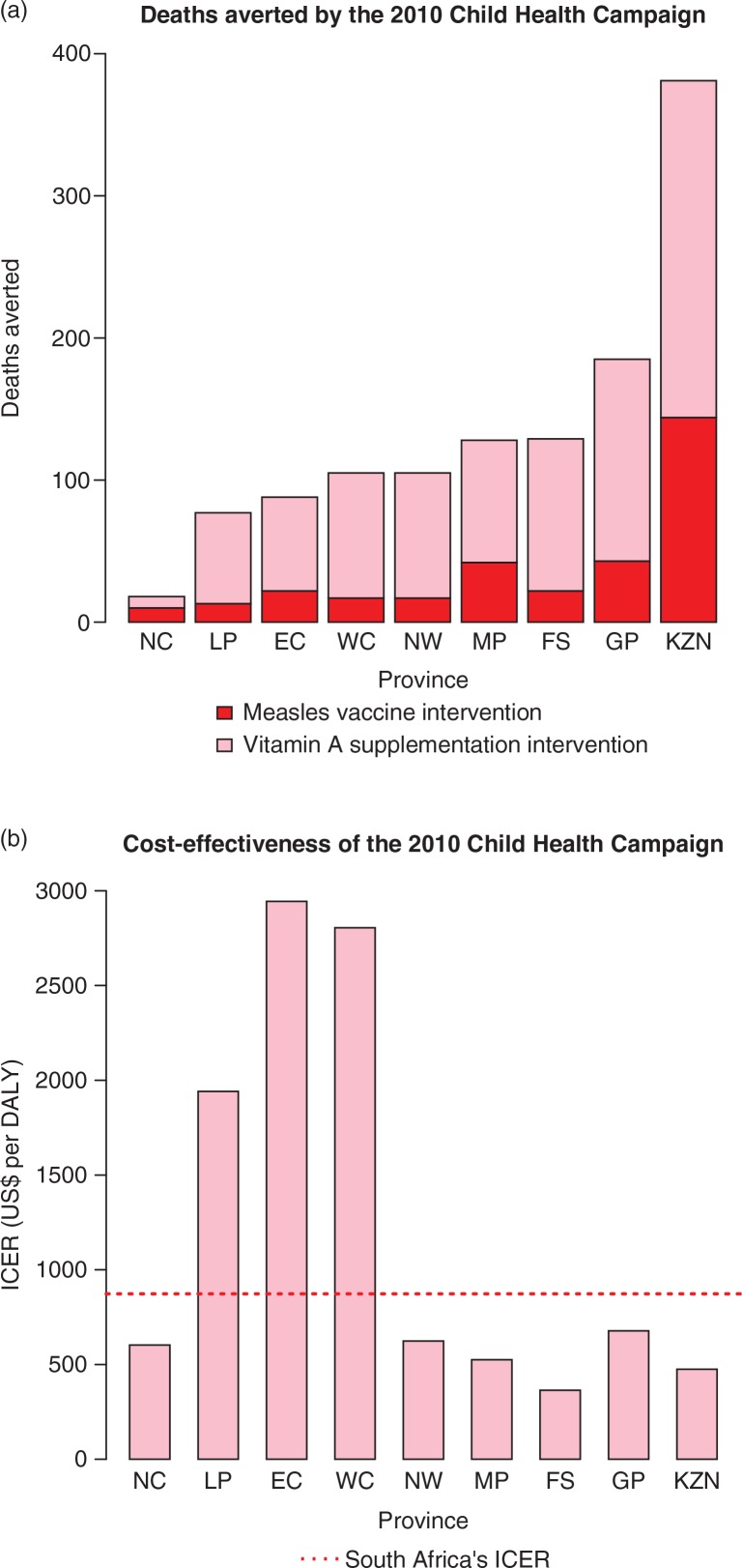
Deaths averted (a) and cost-effectiveness (b) for the 2010 Child Health Campaign by province. EC, Eastern Cape; FS, Free State; GP, Gauteng; KZN, KwaZulu-Natal; LP, Limpopo; MP, Mpumalanga; NC, Northern Cape; NW, North West; WC, Western Cape. ICER, incremental cost-effectiveness ratio (in US$ per DALY, assuming 1 death corresponds to 31 DALYs).

The results of the Poisson regression model (equations 1 and 2) are given in the supplementary data (Table S.1). In this case, the goodness of fit was substantially higher than other models with R^2^=0.94 and RMSE (Root Mean Square Error)=34. Figure 1a and Table 3 present the burden of disease averted. The burden of disease averted by measles vaccination alone was: 327 deaths (95% uncertainty range: 283–371) nationally; the least was 10 deaths averted in the Northern Cape, the most was 144 deaths averted in KwaZulu-Natal. The burden of disease averted by vitamin A supplementation alone was substantial: 818 deaths (95% UR: 672–1,035) nationally; the least was 8 deaths averted in the Northern Cape, the most was 237 deaths averted in KwaZulu-Natal. A combination of high burden of disease and large population reached makes KwaZulu-Natal the greatest beneficiary in terms of burden of disease averted. Overall, the 2010 Child Health Campaign averted 1,145 deaths (92% UR: 994–1,363), including 381 deaths in KwaZulu-Natal and 185 deaths in Gauteng. The lowest number was in the Northern Cape with 18 deaths.

**Table 3:** Burden of disease averted, in deaths averted (95% uncertainty range in parentheses), by the 2010 Child Health Campaign by province and nationally

	EC	FS	GP	KZN	LP	MP	NC	NW	WC	South Africa
Measles vaccination										
	22 (13–32)	22 (13–32)	43 (29–56)	144 (118–172)	13 (7–21)	42 (30–56)	10 (5–16)	17 (10–25)	14 (7–21)	327 (283–371)
Vitamin A supplementation										
	66 (38–115)	107 (53–177)	142 (69–225)	237 (137–376)	64 (35–102)	86 (46–142)	8 (4–13)	88 (51–142)	20 (9–34)	818 (672–1,035)
Campaign (total)										
	88 (57–137)	129 (74–201)	185 (111–271)	381 (275–527)	77 (46–116)	128 (84–186)	18 (11–26)	105 (66–157)	34 (21–51)	1,145 (994–1,363)

EC, Eastern Cape; FS, Free State; GP, Gauteng; KZN, KwaZulu-Natal; LP, Limpopo; MP, Mpumalanga; NC, Northern Cape; NW, North West; WC, Western Cape.

Tables 2 and 4 list the costs and net costs, respectively; Table 4; Fig. 1b give the cost-effectiveness results. The total cost of the platform was $37 million nationally, ranging from about $8 million each in the Eastern Cape and KwaZulu-Natal to $0.5 million in the Northern Cape. The CHC platform's ICER is $27,063 per death averted (95% UR: 18,476–27,063) nationally, ranging from $11,284 per death averted in the Free State to $91,264 per death averted in the Eastern Cape, reflecting the substantial heterogeneity within SA. A combination of high burden of disease, large population reached, and lower costs (about $1.8 million) makes the intervention most cost-effective in the Free State; a combination of lower burden of disease and higher costs (about $8 million) makes the intervention least cost-effective in the Eastern Cape. Gauteng and KwaZulu-Natal present ICERs of $21,018 per death averted and $14,725 per death averted, respectively. These are the two most populous provinces with a high prevalence of inadequate vitamin A status among children (89% in KwaZulu-Natal, 65% in Gauteng) and high burden of diarrhea (13, 14). In the hypothetical scenario, where measles vaccination would be the sole intervention delivered by the CHC platform, the sub ICER would be $88,102 per death averted (95% UR: 64,387–110,174) nationally, ranging from $33,418 per death averted in the Northern Cape, with a smaller population immunized and lower costs, to $367,722 per death averted in the Eastern Cape with higher costs.

**Table 4:** Net costs (in million 2010 US dollars), and incremental cost-effectiveness ratio ICER in US$ per death averted (95% uncertainty range in parentheses) for the 2010 Child Health Campaign by province and nationally

Province	EC	FS	GP	KZN	LP	MP	NC	NW	WC	South Africa
Campaign										
Net costs NC	8.1 (6.7–9.3)	1.5 (0.9–1.8)	3.9 (2.8–4.7)	5.6 (2.6–7.4)	4.7 (3.9–5.5)	2.1 (1.1–2.7)	0.3 (0.1–0.5)	2.1 (1.5–2.4)	2.9 (2.4–3.5)	31.2 (23.1–37.4)
ICER	91,264 (53,847–144,646)	11,284 (5,642–20,739)	21,018 (12,431–35,185)	14,725 (6,324–23,219)	60,171 (37,386–101,742)	16,275 (7,533–26,133)	18,693 (5,208–38,037)	19,344 (10,881–31,775)	86,955 (53,258–147,839)	27,063 (18,476–34,379)
Measles vaccination alone^a^										
Net costs NC_M_	8.0 (6.9–9.1)	1.4 (0.9–1.7)	3.8 (2.9–4.5)	5.2 (2.6–6.6)	4.4 (3.8–4.9)	1.7 (0.8–2.1)	0.2 (<0–0.3)	1.7 (1.3–2.0)	2.5 (2.0–2.8)	28.9 (21.9–33.7)
ICER_M_	367,722 (235,476–629,641)	65,968 (32,891–119,598)	91,698 (56,265–140,399)	39,122 (16,585–50,995)	350,517 (199,888–681,380)	1,598 (16,709–63,333)	33,418 (<0–69,037)	120,745 (55,149–188,635)	209,808 (100,781–380,680)	88,102 (64,387–110,174)

a Hypothetical scenario in which measles vaccination would be the only intervention delivered by the Child Health Campaign.

EC, Eastern Cape; FS, Free State; GP, Gauteng; KZN, KwaZulu-Natal; LP, Limpopo; MP, Mpumalanga; NC, Northern Cape; NW, North West; WC, Western Cape.

**Fig. 2:**
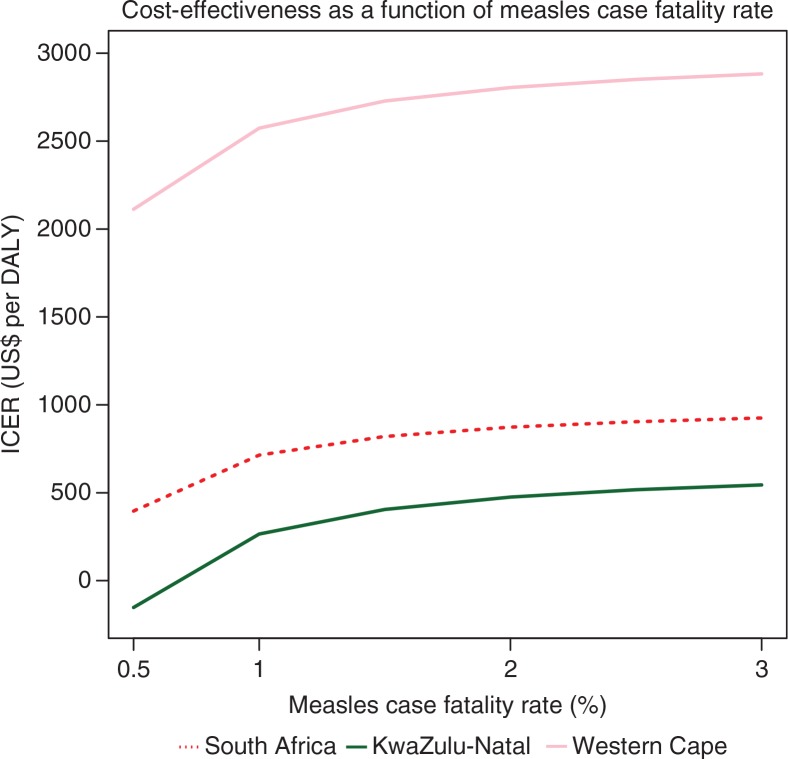
Incremental cost-effectiveness ratio for the 2010 Child Health Campaign nationally, and for KwaZulu-Natal and the Western Cape provinces, as a function of the measles case fatality rate ICER, incremental cost-effectiveness ratio (in US$ per DALY, assuming 1 death corresponds to 31 DALYs).

The extent of the confidence intervals is mainly due to the uncertainty in the number of measles cases averted and related hospitalization costs, a consequence of the values available and used for the measles case fatality rate (CFR). CFRs vary widely between settings, as the criteria of diagnosis are not constant, and measles cases are largely underreported. Measles cases reporting efficiency is also often higher among those hospitalized with complications, which may bias the CFR values (27). Therefore, Fig. 2 plots the 2010 CHC's ICER for South Africa and two provinces (KwaZulu-Natal and the Western Cape) for selected CFR values. Though more sensitive to the lowest value of CFR, overall, the results remained stable: the CHC remains cost-effective nationally (400<ICER<$1,000/DALY); the Western Cape's ICER remains high (>$2,000/DALY) whereas KwaZulu-Natal's ICER remains low (< $600/DALY, with net savings for a CFR of 0.5%).

## Discussion

The 2010 Child Health Campaign delivery platform is cost-effective nationally with a cost-effectiveness of $27,063 per death averted ($873/DALY), but with significant heterogeneity in the results across provinces (Table 4; Fig. 1): cost-effectiveness was best for the Free State ($11,284 per death averted or $364/DALY) and worst for the Eastern Cape ($91,264 per death averted or $2,944/DALY) with a high price tag – about $8 million. These disparities have implications in terms of efficiently allocating resources across provinces. Looking at the hypothetical scenario, where the CHC would only deliver MCV, cost-effectiveness would become worse: $88,102 per death averted ($2,842/DALY) nationally, ranging from $33,418 per death averted ($1,078/DALY) in the Northern Cape to $367,722 per death averted ($11,862/DALY) in the Eastern Cape. Integration of vitamin A supplementation therefore makes the CHC platform more attractive, confirming previous findings (28). The expansion of the platform to vitamin A supplementation substantially increases its economies of scope, with high effectiveness and low cost.

Our numbers differ from a previous study that looked at the cost-effectiveness of measles SIAs alone, in two SA provinces (18). The time horizon and methodology in our study were significantly different: we looked at a one-time comprehensive delivery platform integrating multiple interventions with a shorter time horizon. Our results thus differ from other cost-effectiveness studies on measles SIAs in sub-Saharan African countries, including Uganda and Ethiopia, which also have substantially higher measles burdens (22, 23). These studies looked at the broader cost-effectiveness of measles eradication over a 40-year period (22, 23, 29), and did not consider any other interventions except for measles vaccination. The Child Health Campaign delivery platform appears in the range of high cost interventions in sub-Saharan Africa compared to other interventions in the region against diarrheal diseases (US$500–1,700/DALY), HIV/AIDS (US$600–1,500/DALY), and cardiovascular diseases (US$600–27,000/DALY) (30).

The cost-effectiveness model here presents several limitations. The estimation of burden of disease averted (both morbidity and mortality) is difficult as data on measles and diarrhea incidence and deaths are limited. Hence, epidemiological interactions between measles, diarrhea, and vitamin A deficiency were not incorporated. Measles complications that are serious but considered rare events such as encephalitis (23) were not included. Potential non-specific beneficial effects on survival from immunization (31) were not included because these effects, often estimated upon non-randomized studies, are subject to selection bias (32). The Poisson regression did not model the effectiveness of SIAs against measles that potentially varies with province and time, due to lack of data. The regression model also presents limitations due to the small time series used (1993–2007), which may then be subject to serial correlations. However, we checked the robustness of our findings and found consistent estimates of measles deaths averted when different time lags (other than 7 years) for routine coverage were tried in equation 2. We did not incorporate the full spectrum of helminth-associated morbidity (33), because the population reached with deworming medicines in 2010 was so small that their inclusion would not affect results. In relation to costs, we had an incomplete recording of projected costs and expenditure on costs was not available. However, projected costs from campaign planners provide a reasonable estimate. Also, we did not discount health benefits and costs over the 3 years, as this would not greatly affect results over a 3-year time frame (34). Finally, polio and measles vaccination interventions may be considered global goods. In the larger context of polio eradication (35) and measles elimination (22, 23), eradication not only reduces infections but also eliminates the need for future vaccinations. However, our analysis uses sub-national data toward describing the SA situation, a step toward these global goods.

These results have important implications for SA's decision makers in terms of how to optimize the current Child Health Campaign platform. SA's routine health services are currently free of charge to all children 6 years and under. Spending US$37 million on a nationwide campaign may not be the best allocation of scarce government resources given that child mortality is distributed heterogeneously in the country. As shown on Fig. 1, a campaign selectively targeting the provinces of KwaZulu-Natal, Gauteng, the Free State, Mpumalanga, and North West may be more economically attractive while addressing up to 80% of the burden of disease that can be averted by a nationwide CHC. A comparison of campaign costs with the costs and projected benefits (from improved coverage) of expanding routine child health services in areas where they are especially weak should also be made. In the comparison, the current performance of the SA health system must be considered, as routine immunization coverage is widely heterogeneous among districts, with some districts, particularly in KwaZulu-Natal, presenting low immunization rates (36). In addition, routine activities rarely achieve universal coverage especially when the population is diverse and remote rural areas are difficult to reach. Delivery platforms periodically delivered directly to communities such as Child Health Campaigns may raise overall coverage and reduce coverage heterogeneity, while targeting those not reached by inadequate routine health services (37–39). Child Health Campaigns may be therefore required for equitable access to basic child health services, and can also be implemented in conjunction with WHO's ‘Reaching Every District’ strategy which focuses on building national capacity from district level upward to maximize universal vaccine access (40).

Further work in sub-Saharan Africa could determine country-specific cost-effectiveness of Child Health Campaign expansion, and which combination of child health interventions and which size and groups of beneficiaries, it is optimal to target. In particular, there could be an emphasis on the economies of scope, including the estimation of which costs are apportioned to which interventions (not available for this paper), when there is expansion of the delivery platform. Important considerations are the burden of disease, as well as issues of health rights and entitlements in targeting children who are often overlooked by the health system. Our approach to economic evaluation in SA can be used to evaluate other countries’ Child Health Campaign platforms. It could aid Ministries of Health at both national and provincial level in designing a Child Health Campaign platform that achieves better outcomes at lower costs: it enables a comparison of tradeoffs with other delivery platforms, such as routine immunization services. In particular, there may be a transitional period when a particular country moves away from a CHC platform to deliver health care toward strengthening its routine health services. Finally, our approach emphasizes the consideration of health system delivery platforms and the importance of integrating and combining multiple interventions onto the same delivery platform.

## Footnotes

1 The number of measles deaths averted by the CHC platform is estimated at the provincial level, though the Poisson regression model is estimated at the national level, as data on measles-related deaths were available at the national level only.

2 Only one time lag (i.e. 7 years) was used for the routine coverage covariate because its time series presents substantial correlation.
